# SG-APSIC1080: Surveillance and control efforts for carbapenemase-producing gram-negative bacteria at a high-burden tertiary-care healthcare facility in Ho Chi Minh City, Vietnam

**DOI:** 10.1017/ash.2023.69

**Published:** 2023-03-16

**Authors:** Tuan Huynh, Lan Pham, Loan Luong, Tuan Le, Khanh Lě, Duyen Bui, Truc Ta, Thoa Trinh, Yen Nguyen, Diep Bui, Nga Vo, Nga Nguyen, Bao Nguyen, Binh Truong

**Affiliations:** University Medical Center, Ho Chi Minh City, Vietnam; University Medical Center, Ho Chi Minh City, Vietnam; University Medical Center, Ho Chi Minh City, Vietnam; University Medical Center, Ho Chi Minh City, Vietnam; University Medical Center, Ho Chi Minh City, Vietnam; University Medical Center, Ho Chi Minh City, Vietnam; University Medical Center, Ho Chi Minh City, Vietnam; University Medical Center, Ho Chi Minh City, Vietnam; University Medical Center, Ho Chi Minh City, Vietnam; University of Medicine and Pharmacy, Ho Chi Minh City, Vietnam; University of Medicine and Pharmacy, Ho Chi Minh City, Vietnam; PATH, Hanoi, Vietnam; University Medical Center and University of Medicine and Pharmacy, Ho Chi Minh City, Vietnam; University Medical Center and University of Medicine and Pharmacy, Ho Chi Minh City, Vietnam

## Abstract

**Objectives:** In Vietnam, although surveillance and control of multidrug-resistant organisms is a national priority, information on the burden of these pathogens remains scarce. At the University Medical Center in Ho Chi Minh City, we assessed the proportion of carbapanemase-producing carbapenem-resistant organisms (CP-CRO) and evaluated an intervention package to prevent transmission of carbapenemase-producing carbapenem-resistant Enterobacterieacea (CP-CRE) in the intensive care unit (ICU). **Methods:** All gram-negative isolates collected between November 2018 to April 2019 were tested for carbapenem resistance using the disc-diffusion method. Carbapenem-resistant bacteria, defined as meropenem resistant, were tested for phenotypic carbapenemase-production using the Becton Dickinson Phoenix CPO Detect assay. An intervention package, including placement of patients in cohorts, enhanced barrier precautions, enhanced discharge environmental cleaning, and CP-CRE rectal screening, was implemented from July 2019 through December 2020. During this period, all ICU patients were screened on admission, and negative patients were rescreened every 2 days or 7 days until discharge, death, or CRE-positive result. Admission prevalence and incidence of CP-CRE transmission was calculated among CP-CRE infected or colonized patients. **Results:** Among 599 gram-negative isolates collected, 108 were carbapenem-resistant isolates, of which 107 (99%) were CP-CRO by the phenotypic method. Most CP-CRO were *Acinetobacter baumannii* (42%) and *Klebsiella pneumoniae* (36%). Of 1,206 patients, 433 (35.9%) were already colonized or infected with CP-CRE before admission to the ICU. The incidence rate (cases per 100 risk days) of CP-CRE colonization or infection during ICU treatment decreased from 11.5 before the intervention to 2.9 after the implementation of the intervention package. The average number of days to change from a negative to positive screening result in the intervention phase was 7.4, compared with 4.9 days during preintervention phase. **Conclusions:** Nearly all CROs isolated from our ICU are carbapenemase-producing CROs, with high presence on admission as well as new acquisition during an ICU stay. An intervention package containing enhanced infection control measures was effective in reducing CP-CRE transmission.

